# Supplementary material for: Exploring Sex Differences of Beta-Blockers in the Treatment of Hypertension: A Systematic Review and Meta-Analysis
Source: Biomedicines. 2023 May 22;11(5):1494. doi: 10.3390/biomedicines11051494 (PMC10216365; doi:10.3390/biomedicines11051494)
Supplement: Supplementary file 1 [file biomedicines-11-01494-s001.zip › biomedicines-2373889-supplementary.pdf]

Table S1 Literature search: strategy for PubMed (NCBI) and Embase (Ovid) databases

Search PubMed	Search Embase
**Component 1: Antihypertensive medication:** "diuretics"[Mesh] OR "adrenergic beta-antagonists"[Mesh] OR “beta blockers” [Title/Abstract] OR "Antihypertensive agents"[Mesh] OR “blood pressure lowering therapy” [Title/Abstract] OR “antihypertensive medication” [Title/Abstract] OR “antihypertensive therapy” [Title/Abstract] OR "angiotensin-converting enzyme inhibitors"[Mesh] OR “ACE inhibitors” [Title/Abstract] OR "Angiotensin receptor antagonists"[Mesh] OR “angiotensin receptor blockers” [Title/Abstract] OR "sympatholytics"[Mesh]OR "Calcium Channel Blockers"[Mesh]	**Component 1: Antihypertensive medication** exp diuretic agent/ or exp beta adrenergic receptor blocking agent/ or exp adrenergic receptor blocking agent/ or exp antihypertensive agent/ or exp dipeptidyl carboxypeptidase inhibitor/ or exp angiotensin receptor antagonist/ or exp calcium channel blocking agent.ti,ab.
**Component 2: Cardiac geometry:** "ventricular remodeling"[Mesh] OR “ventricular remodeling” [Title/Abstract] OR “cardiac remodeling” [Title/Abstract] OR “cardiac adaptation” [Title/Abstract] OR “LV geometry” [Title/Abstract] OR “left ventricular geometry” [Title/Abstract] OR “cardiac geometry” [Title/Abstract] OR “cardiac dimension” [Title/Abstract] OR "left ventricle remodeling "[Title/Abstract] OR " Hypertrophy, Left Ventricular "[Mesh] OR “left ventricular hypertrophy” [Title/Abstract] OR "echocardiography"[Mesh] OR Echocardiography [Title/Abstract] OR “left ventricular mass” [Title/Abstract] OR “left ventricular mass index” [Title/Abstract] OR “relative wall thickness” [Title/Abstract] OR “concentric cardiac remodeling” [Title/Abstract] OR “eccentric cardiac remodeling” [Title/Abstract]	**Component 2: Cardiac geometry** exp heart ventricle remodeling/ or (ventricular remodeling or cardiac remodeling or cardiac adaptation or LV geometry or left ventricular remodeling or cardiac geometry or cardiac dimension).ti,ab. or exp echocardiography/ or echocardiography.ti,ab.
**Component 3: Heart failure:** "Heart Failure"[Mesh] OR "Heart Failure, Systolic "[Mesh]	**Component 3: Heart failure** exp heart failure.ti,ab.
**Component 4: Diastolic dysfunction:** “heart failure, diastolic”[Mesh] OR “diastolic dysfunction” [Title/Abstract]	**Component 4: Diastolic dysfunction** exp diastolic dysfunction/ or diastolic function.ti,ab.
**Component 5: Myocardial infarction:** "myocardial infarction" [Mesh] OR “myocardial infarction” [Title/Abstract] OR “acute myocardial infarction” [Title/Abstract] OR “heart attack” [Title/Abstract]	**Component 5: Myocardial infarction** exp heart infarction.ti,ab.

**Component 6: CVA**: Stroke [Mesh] OR “cerebrovascular accident” [Title/Abstract] OR “acute cerebrovascular accident” [Title/Abstract] OR “acute cerebrovascular insult” [Title/Abstract]	**Component 6: CVA**<br>exp cerebrovascular accident.ti,ab.

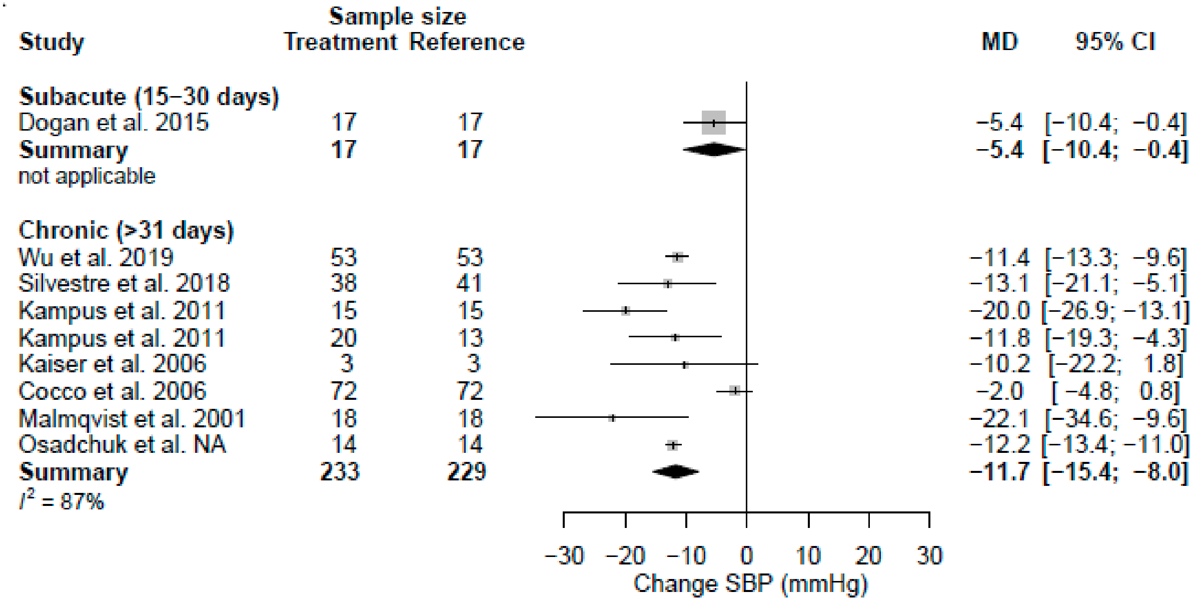

Figure S1 Forest plot of systolic blood pressure (SBP) change in mmHg after sub-acute and chronic beta-blocker use compared to baseline for females. MD = mean difference.

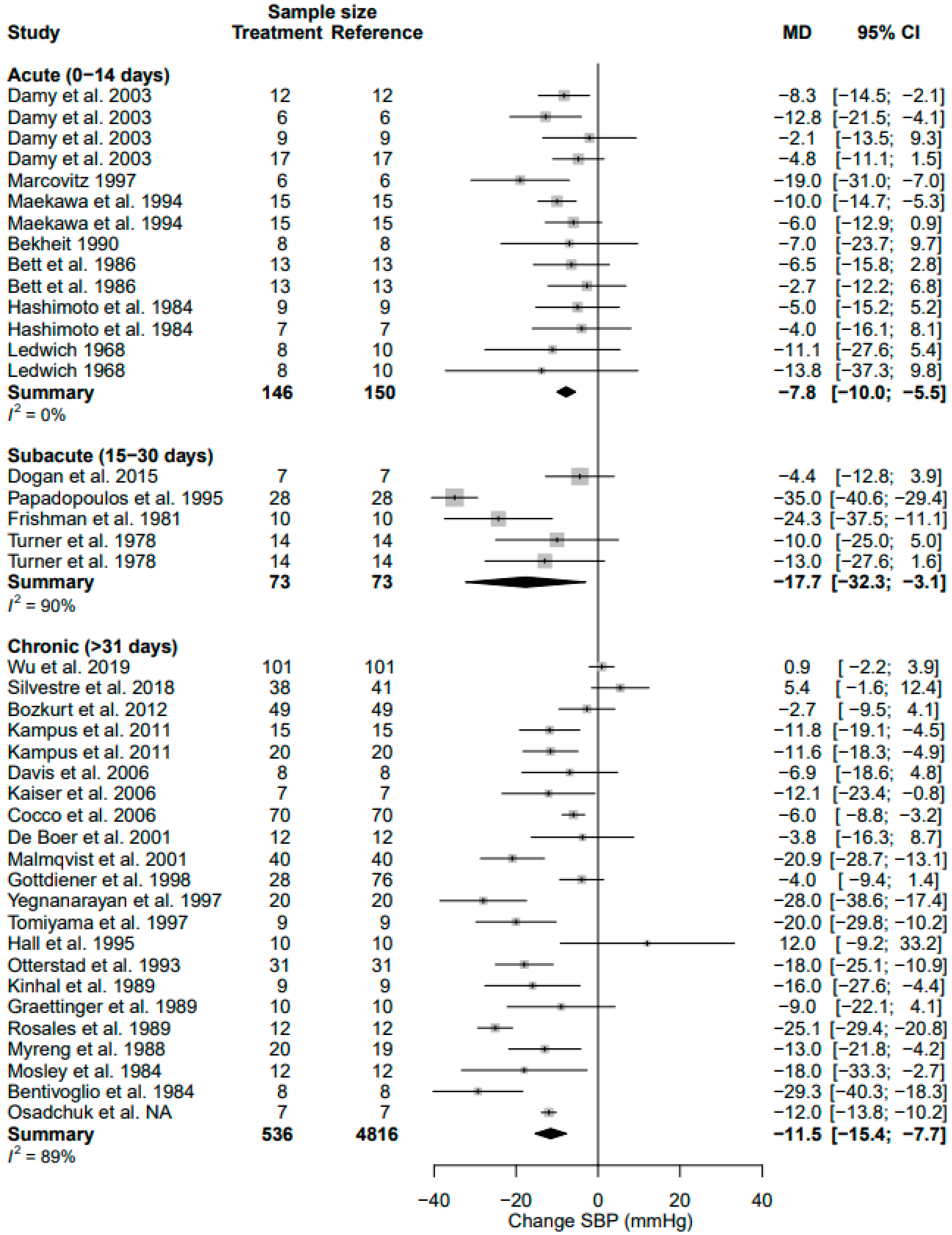

Figure S2 Forest plot of systolic blood pressure (SBP) change in mmHg after acute, sub-acute and chronic beta-blocker use compared to baseline for males. MD = mean difference.

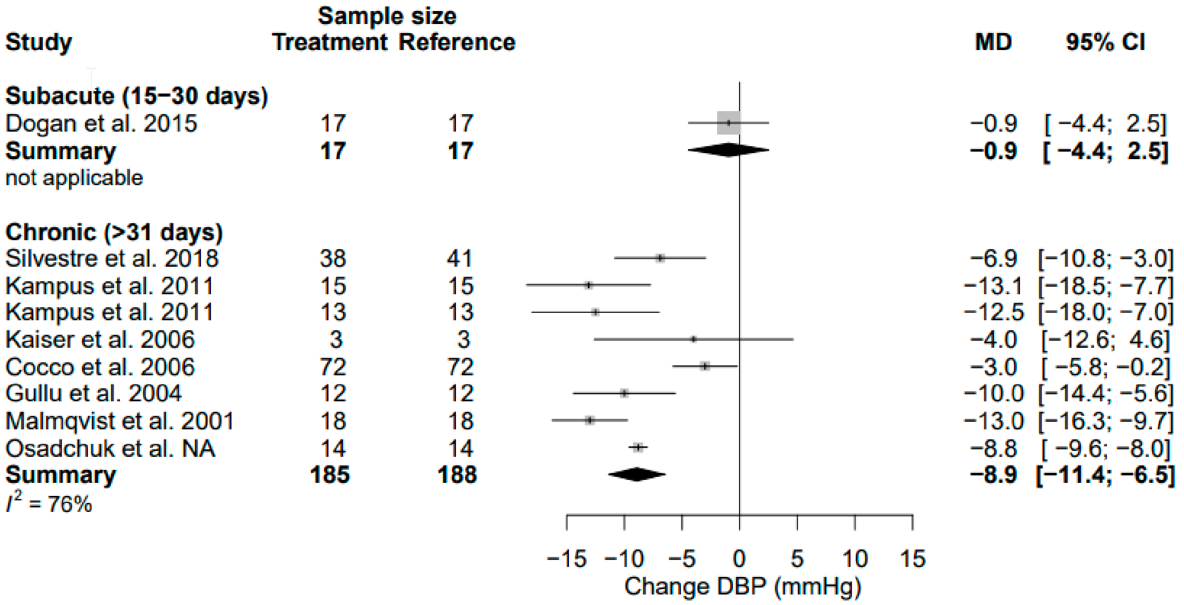

Figure S3 Forest plot of diastolic blood pressure (DBP) change in mmHg after sub-acute and chronic beta-blocker use compared to baseline for females. MD = mean difference.

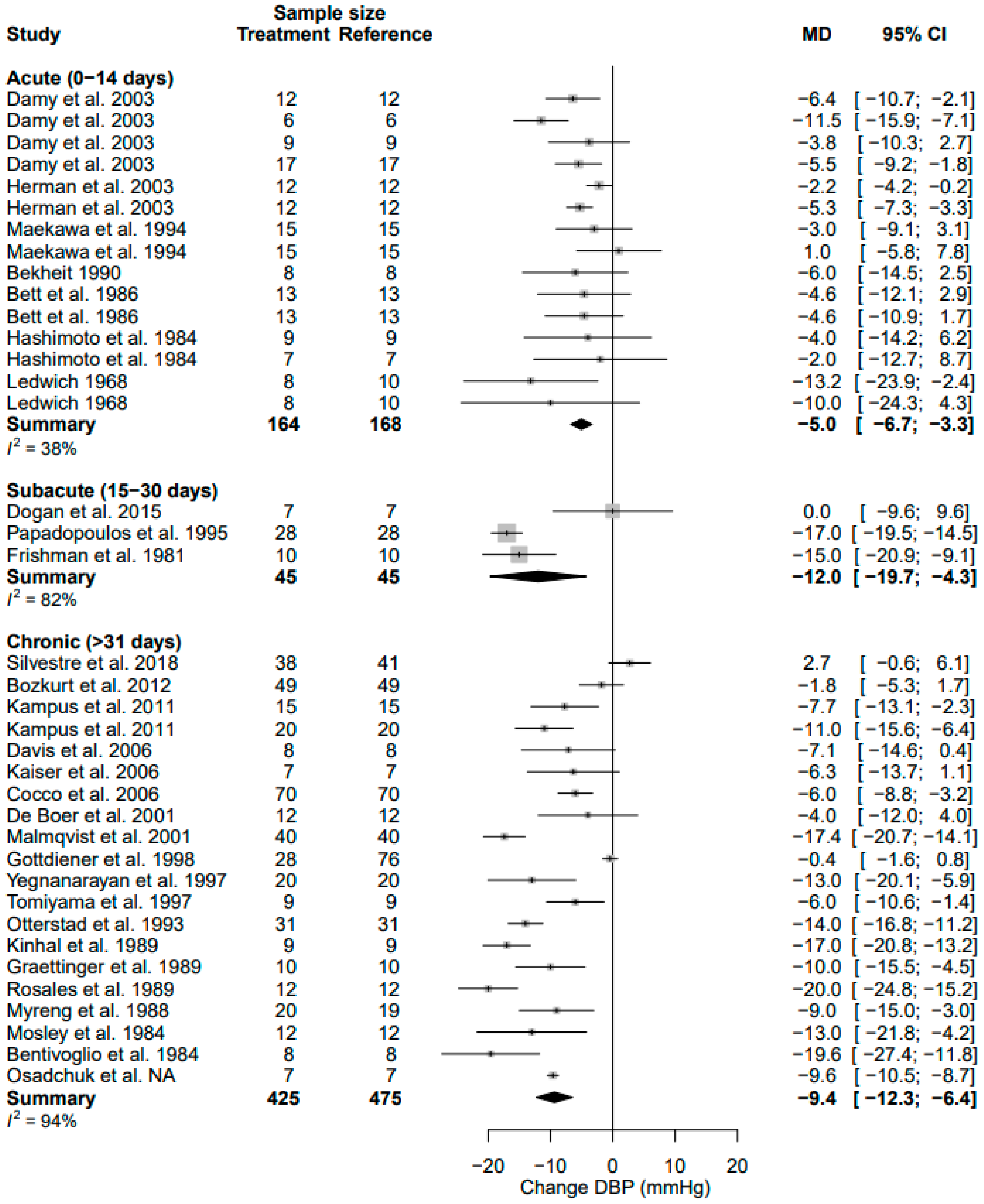

Figure S4 Forest plot of diastolic blood pressure (DBP) change in mmHg after acute, sub-acute and chronic beta-blocker use compared to baseline for males. MD = mean difference.

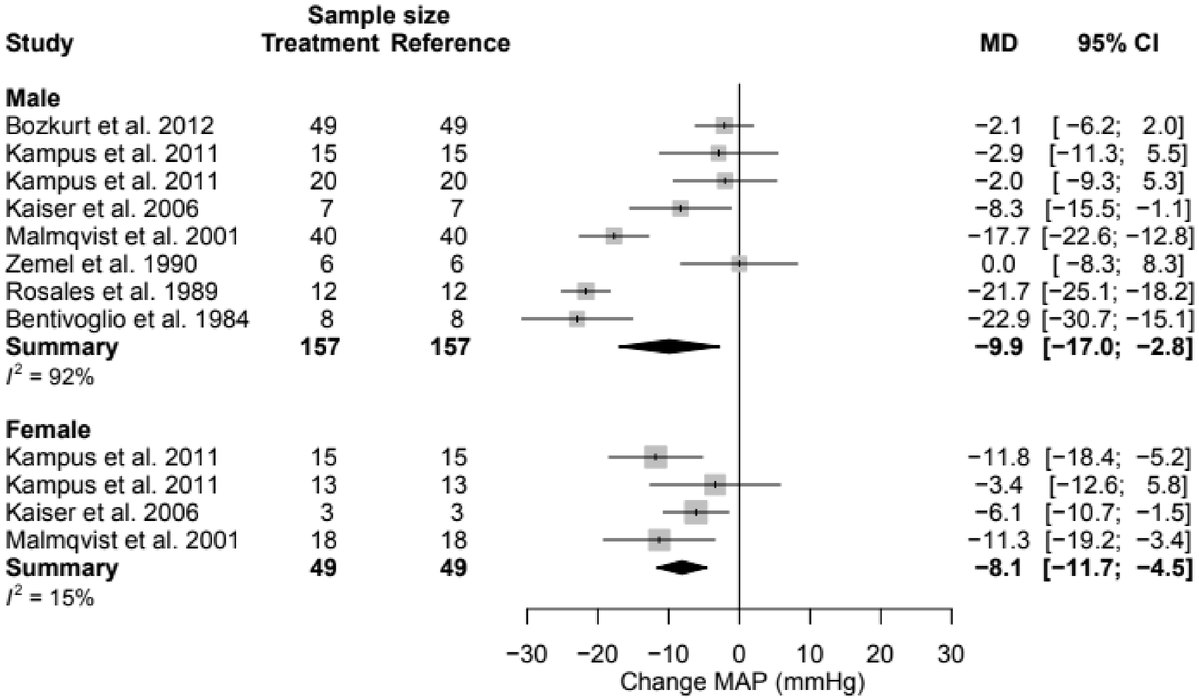

Figure S5 Forest plot of mean arterial pressure (MAP) change in mmHg after beta-blocker use compared to baseline for females and males. MD = mean difference.

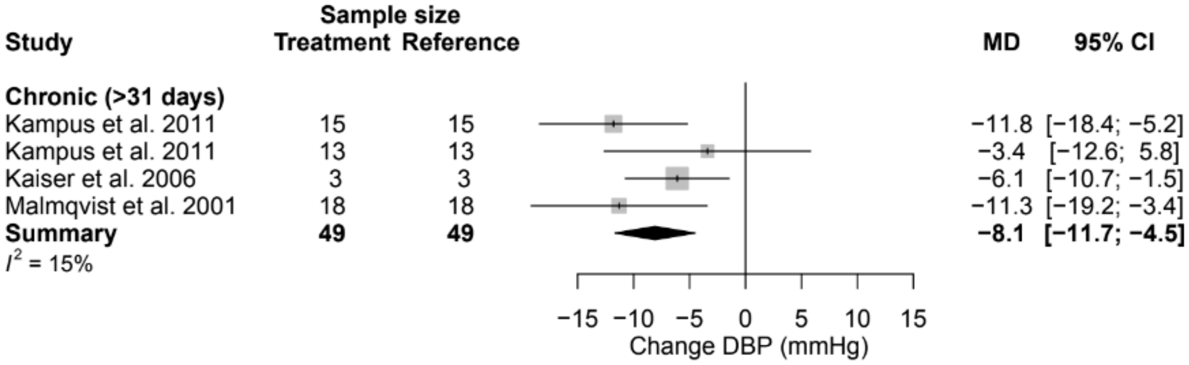

Figure S6 Forest plot of mean arterial pressure (MAP) change in mmHg after acute, sub-acute and chronic beta-blocker use compared to baseline for females. MD = mean difference.

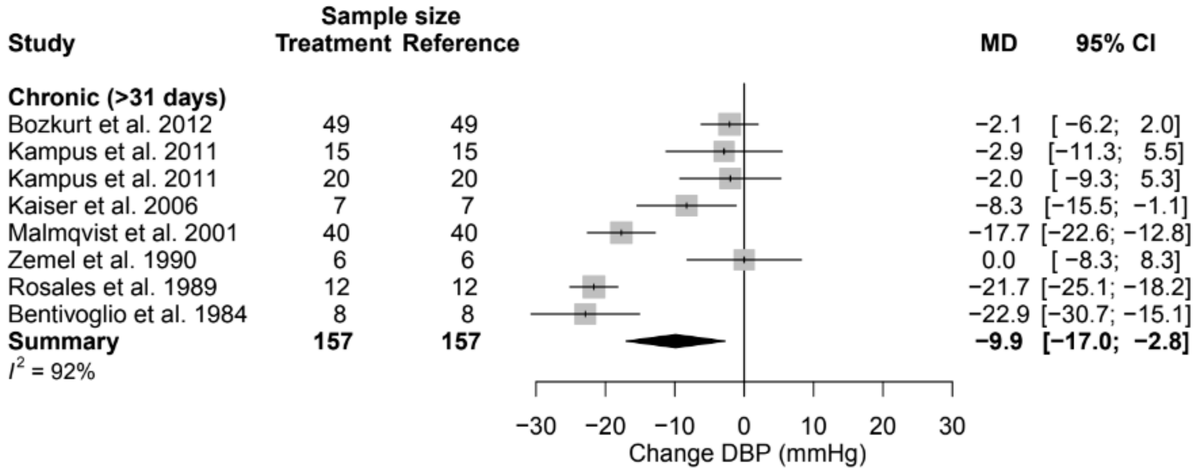

Figure S7 Forest plot of mean arterial pressure (MAP) change in mmHg after acute, sub-acute and chronic beta-blocker use compared to baseline for males. MD = mean difference.

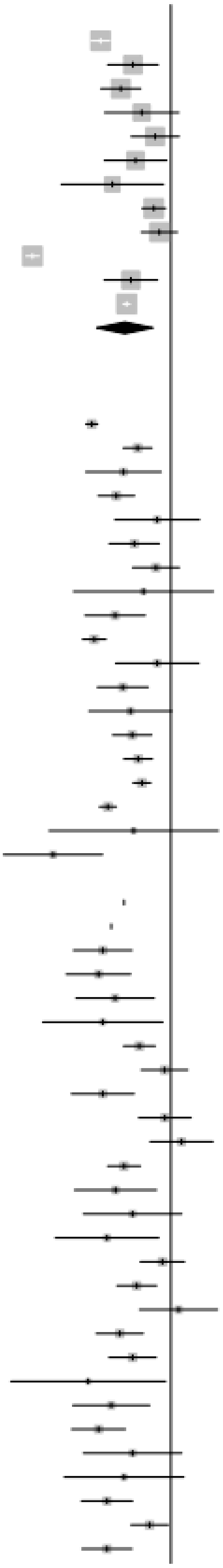

Study	Sample size Treatment	Sample size Reference	MD	95% CI
**Female**				
Wu et al. 2019	53	53	−16.5	[−18.6; −14.4]
Silvestre et al. 2018	38	41	−8.9	[−14.8; −3.0]
Dogan et al. 2015	17	17	−11.8	[−16.5; −7.1]
Yeoh et al. 2011	7	7	−6.8	[−15.6; 1.9]
Kampus et al. 2011	15	15	−3.7	[−9.3; 1.9]
Kampus et al. 2011	13	13	−8.3	[−15.6; −1.0]
Ito et al. 2009	12	12	−13.7	[−25.8; −1.7]
Cocco et al. 2006	72	72	−4.0	[−6.8; −1.2]
Kaiser et al. 2006	3	3	−2.7	[−6.9; 1.5]
Taniguchi et al. 2003	2	2	−32.5	[−34.0; −31.0]
Malmqvist et al. 2001	18	18	−9.4	[−15.7; −3.1]
Osadchuk et al. NA	14	14	−10.4	[−11.3; −9.5]
**Summary**	**264**	**267**	**−10.8**	**[−17.4; −4.2]**
I^2 = 98%				
**Male**				
Wu et al. 2019	101	101	−18.6	[−20.0; −17.2]
Silvestre et al. 2018	38	41	−7.8	[−11.2; −4.4]
Dogan et al. 2015	7	7	−11.1	[−20.0; −2.3]
Bozkurt et al. 2012	49	49	−12.8	[−17.1; −8.5]
Yeoh et al. 2011	9	9	−3.2	[−13.2; 6.7]
Kampus et al. 2011	15	15	−8.6	[−14.5; −2.7]
Kampus et al. 2011	20	20	−3.5	[−9.0; 2.0]
Ito et al. 2009	7	7	−6.4	[−22.9; 10.0]
Davis et al. 2006	8	8	−13.1	[−20.2; −6.0]
Cocco et al. 2006	70	70	−18.0	[−20.8; −15.2]
Kaiser et al. 2006	7	7	−3.2	[−13.0; 6.6]
Damy et al. 2003	12	12	−11.3	[−17.3; −5.3]
Damy et al. 2003	6	6	−9.5	[−19.2; 0.2]
Damy et al. 2003	9	9	−9.1	[−13.7; −4.5]
Damy et al. 2003	17	17	−7.7	[−11.1; −4.3]
Herman et al. 2003	12	12	−6.8	[−8.9; −4.7]
Herman et al. 2003	12	12	−14.8	[−16.8; −12.8]
Taniguchi et al. 2003	4	4	−8.8	[−28.6; 11.1]
Taniguchi et al. 2003	3	3	−27.7	[−39.3; −16.0]
Taniguchi et al. 2003	1	1	−46.0	
Taniguchi et al. 2003	1	1	−11.0	
Taniguchi et al. 2003	1	1	−14.0	
Ridha et al. 2002	15	15	−16.0	[−22.9; −9.1]
Bennett et al. 2002	12	12	−17.0	[−24.6; −9.4]
De Boer et al. 2001	12	12	−13.1	[−22.3; −3.9]
Kaye et al. 2001	10	10	−16.0	[−30.1; −1.9]
Malmqvist et al. 2001	40	40	−7.4	[−11.1; −3.7]
Gottdiener et al. 1998	28	76	−1.5	[−7.0; 4.0]
Marcovitz et al. 1997	6	6	−16.0	[−23.4; −8.6]
Silke et al. 1997	9	9	−1.4	[−7.6; 4.8]
Silke et al. 1997	9	9	2.5	[−4.9; 9.9]
Papadopoulos et al. 1995	28	28	−11.0	[−14.9; −7.1]
Hall et al. 1995	14	14	−13.0	[−22.6; −3.4]
Heesch et al. 1995	15	15	−9.0	[−20.6; 2.6]
Heesch et al. 1995	15	15	−15.0	[−27.2; −2.8]
Maekawa et al. 1994	15	15	−2.0	[−7.2; 3.2]
Maekawa et al. 1994	15	15	−8.0	[−12.7; −3.3]
Ishida et al. 1993	6	9	1.8	[−7.4; 11.0]
Otterstad et al. 1993	31	31	−12.0	[−17.5; −6.5]
Kyriakides et al. 1992	28	28	−9.0	[−14.6; −3.4]
Bekheit 1990	8	8	−19.4	[−37.6; −1.2]
Brune et al. 1990	10	10	−14.0	[−23.0; −5.0]
Todd et al. 1990	20	20	−17.0	[−23.3; −10.7]
Eichhorn et al. 1990	15	15	−9.0	[−20.6; 2.6]
Kronenberg et al. 1990	10	10	−11.0	[−25.1; 3.1]
Kinhal et al. 1989	9	9	−15.0	[−21.0; −9.0]
Graettinger et al. 1989	10	10	−5.0	[−9.4; −0.6]
Myreng et al. 1988	20	20	−15.0	[−20.9; −9.1]

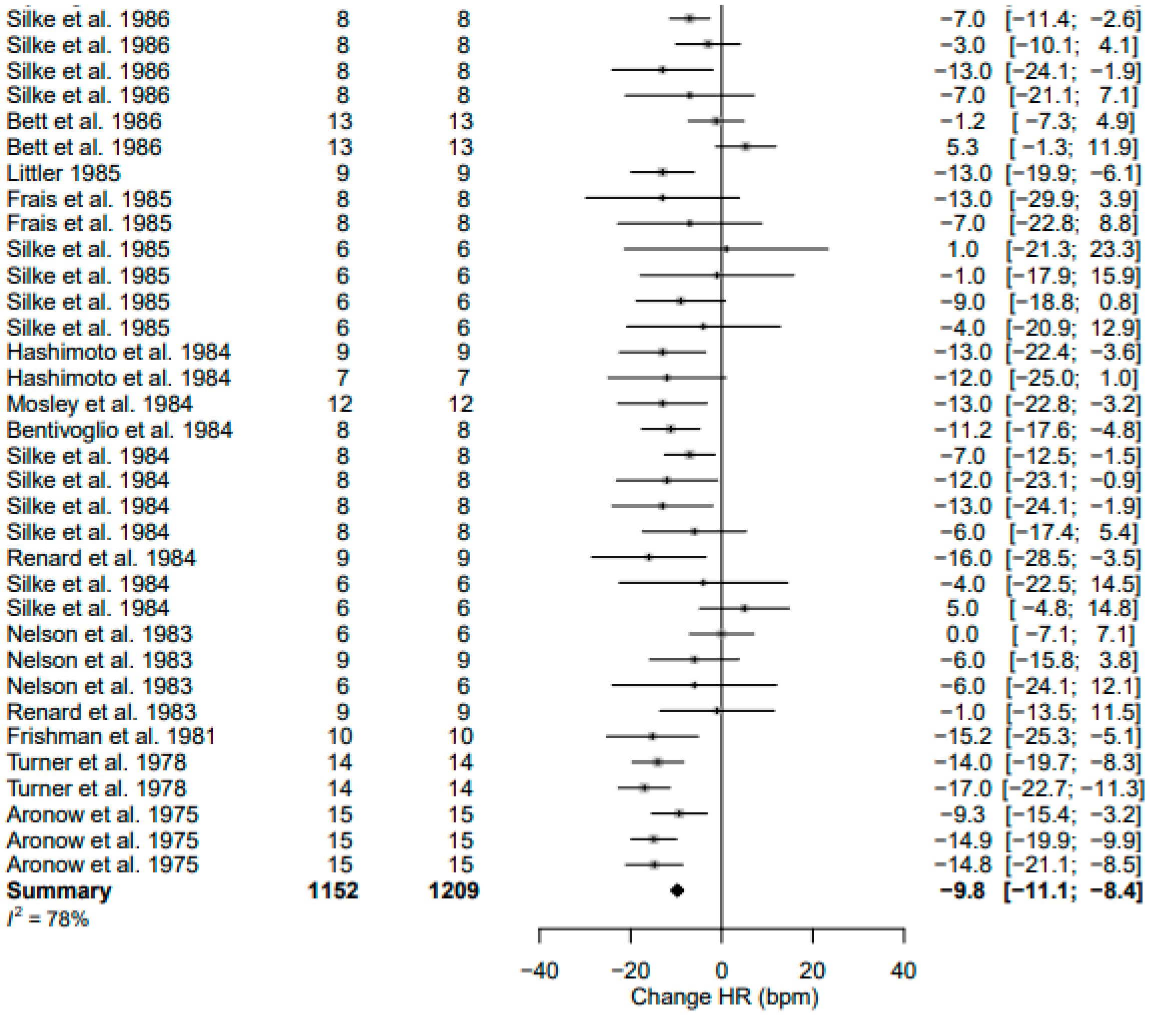

Figure S8 Forest plot of heart rate (HR) change in beats per minute (bpm) after beta-blocker use compared to baseline for females and males. MD = mean difference.

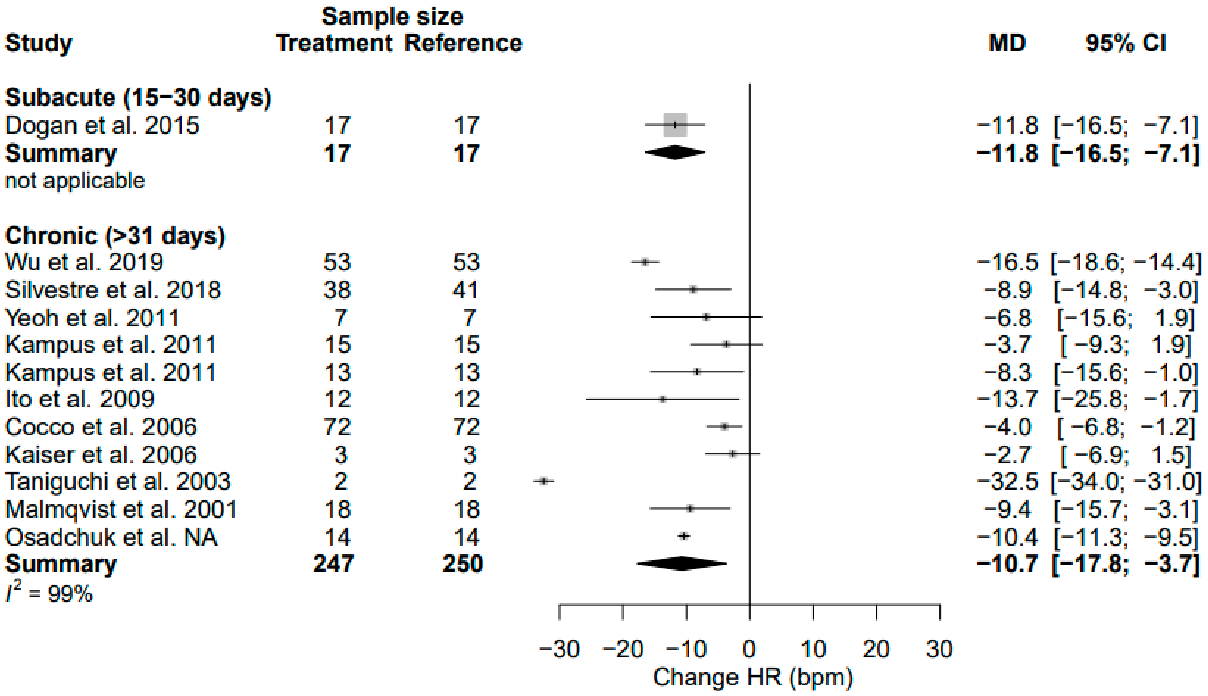

Figure S9 Forest plot of heart rate (HR) change in beats per minute (bpm) after sub-acute and chronic be-ta-blocker use compared to baseline for females. MD = mean difference.

Study	Sample size Treatment	Sample size Reference	MD	95% CI
**Acute (0−14 days)**				
Damy et al. 2003	12	12	−11.3	[−17.3; −5.3]
Damy et al. 2003	6	6	−9.5	[−19.2; 0.2]
Damy et al. 2003	9	9	−9.1	[−13.7; −4.5]
Damy et al. 2003	17	17	−7.7	[−11.1; −4.3]
Herman et al. 2003	12	12	−6.8	[−8.9; −4.7]
Herman et al. 2003	12	12	−14.8	[−16.8; −12.8]
Marcovitz et al. 1997	6	6	−16.0	[−23.4; −8.6]
Silke et al. 1997	9	9	−1.4	[−7.6; 4.8]
Silke et al. 1997	9	9	2.5	[−4.9; 9.9]
Maekawa et al. 1994	15	15	−2.0	[−7.2; 3.2]
Maekawa et al. 1994	15	15	−8.0	[−12.7; −3.3]
Kyriakides et al. 1992	28	28	−9.0	[−14.6; −3.4]
Bekheit 1990	8	8	−19.4	[−37.6; −1.2]
Brune et al. 1990	10	10	−14.0	[−23.0; −5.0]
Todd et al. 1990	20	20	−17.0	[−23.3; −10.7]
Kronenberg et al. 1990	10	10	−11.0	[−25.1; 3.1]
Silke et al. 1986	8	8	−7.0	[−11.4; −2.6]
Silke et al. 1986	8	8	−3.0	[−10.1; 4.1]
Silke et al. 1986	8	8	−13.0	[−24.1; −1.9]
Silke et al. 1986	8	8	−7.0	[−21.1; 7.1]
Bett et al. 1986	13	13	−1.2	[−7.3; 4.9]
Bett et al. 1986	13	13	5.3	[−1.3; 11.9]
Frais et al. 1985	8	8	−13.0	[−29.9; 3.9]
Frais et al. 1985	8	8	−7.0	[−22.8; 8.8]
Silke et al. 1985	6	6	1.0	[−21.3; 23.3]
Silke et al. 1985	6	6	−1.0	[−17.9; 15.9]
Silke et al. 1985	6	6	−9.0	[−18.8; 0.8]
Silke et al. 1985	6	6	−4.0	[−20.9; 12.9]
Hashimoto et al. 1984	9	9	−13.0	[−22.4; −3.6]
Hashimoto et al. 1984	7	7	−12.0	[−25.0; 1.0]
Silke et al. 1984	8	8	−7.0	[−12.5; −1.5]
Silke et al. 1984	8	8	−12.0	[−23.1; −0.9]
Silke et al. 1984	8	8	−13.0	[−24.1; −1.9]
Silke et al. 1984	8	8	−6.0	[−17.4; 5.4]
Renard et al. 1984	9	9	−16.0	[−28.5; −3.5]
Silke et al. 1984	6	6	−4.0	[−22.5; 14.5]
Silke et al. 1984	6	6	5.0	[−4.8; 14.8]
Nelson et al. 1983	6	6	0.0	[−7.1; 7.1]
Nelson et al. 1983	9	9	−6.0	[−15.8; 3.8]
Nelson et al. 1983	6	6	−6.0	[−24.1; 12.1]
Renard et al. 1983	9	9	−1.0	[−13.5; 11.5]
Aronow et al. 1975	15	15	−9.3	[−15.4; −3.2]
Aronow et al. 1975	15	15	−14.9	[−19.9; −9.9]
Aronow et al. 1975	15	15	−14.8	[−21.1; −8.5]
Ledwich 1968	9	10	−14.3	[−30.4; 1.9]
Ledwich 1968	8	10	−30.1	[−44.3; −15.9]
**Summary**	**457**	**460**	**−8.2**	**[−10.1; −6.4]**
I^2 = 68%				
**Subacute (15−30 days)**				
Dogan et al. 2015	7	7	−11.1	[−20.0; −2.3]
Papadopoulos et al. 1995	28	28	−11.0	[−14.9; −7.1]
Frishman et al. 1981	10	10	−15.2	[−25.3; −5.1]
Turner et al. 1978	14	14	−14.0	[−19.7; −8.3]
Turner et al. 1978	14	14	−17.0	[−22.7; −11.3]
**Summary**	**73**	**73**	**−13.1**	**[−15.7; −10.6]**
I^2 = 0%				

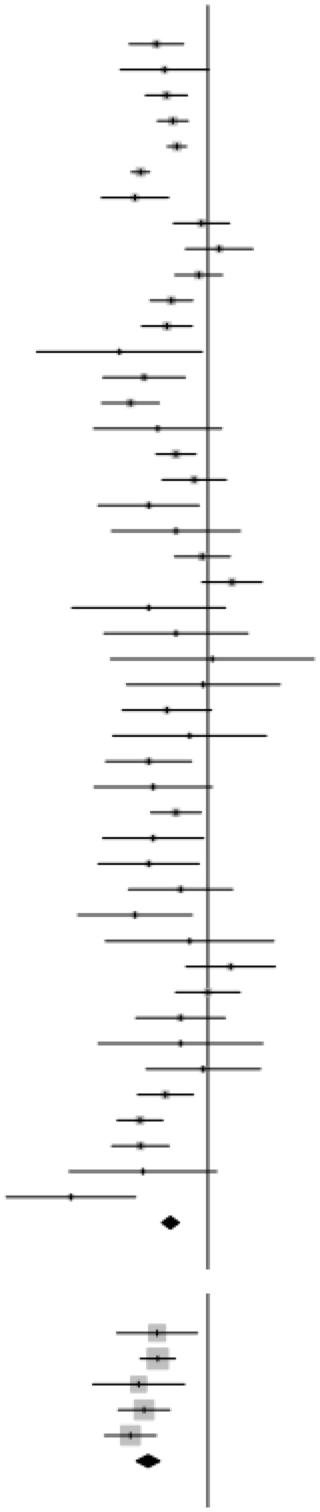

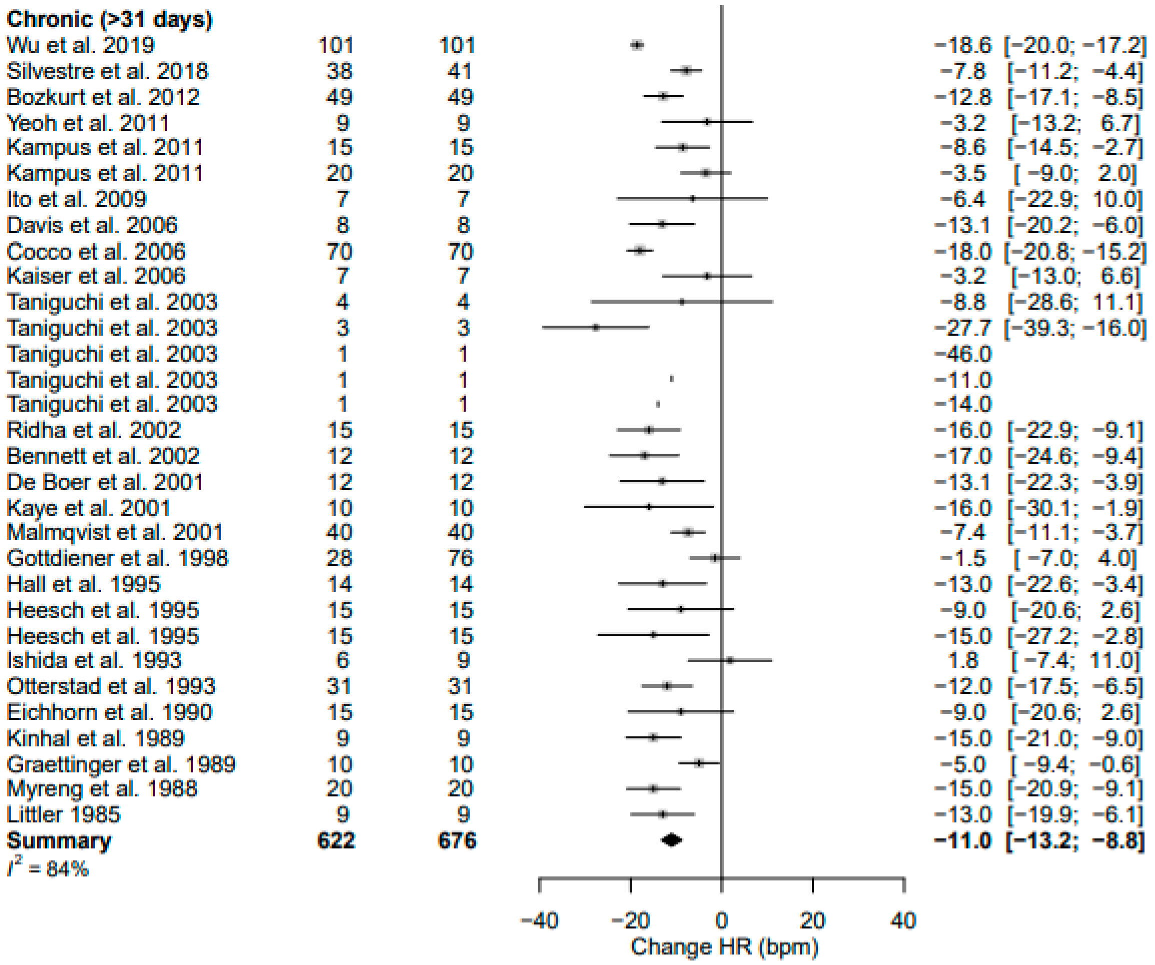

Figure S10 Forest plot of heart rate (HR) change in beat per minute (bpm) after acute, sub-acute and chronic beta-blocker use compared to baseline for males. MD = mean difference.

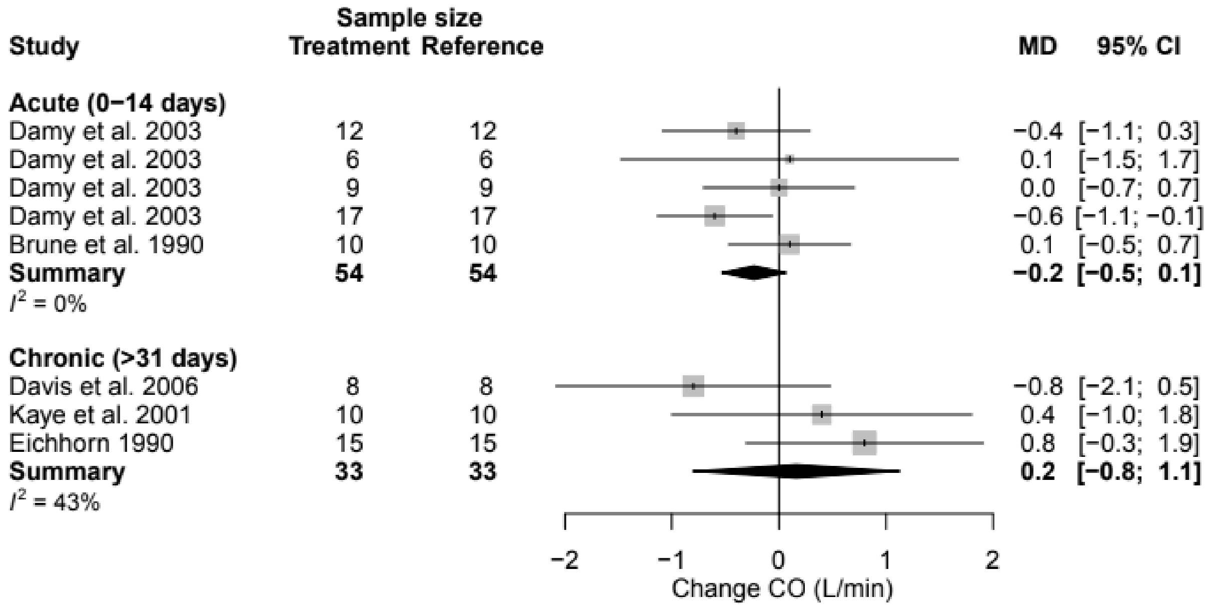

Figure S11 Forest plot of cardiac output (CO) change in liter per minute (L/min) after acute and chronic be-ta-blocker use compared to baseline for males. MD = mean difference.

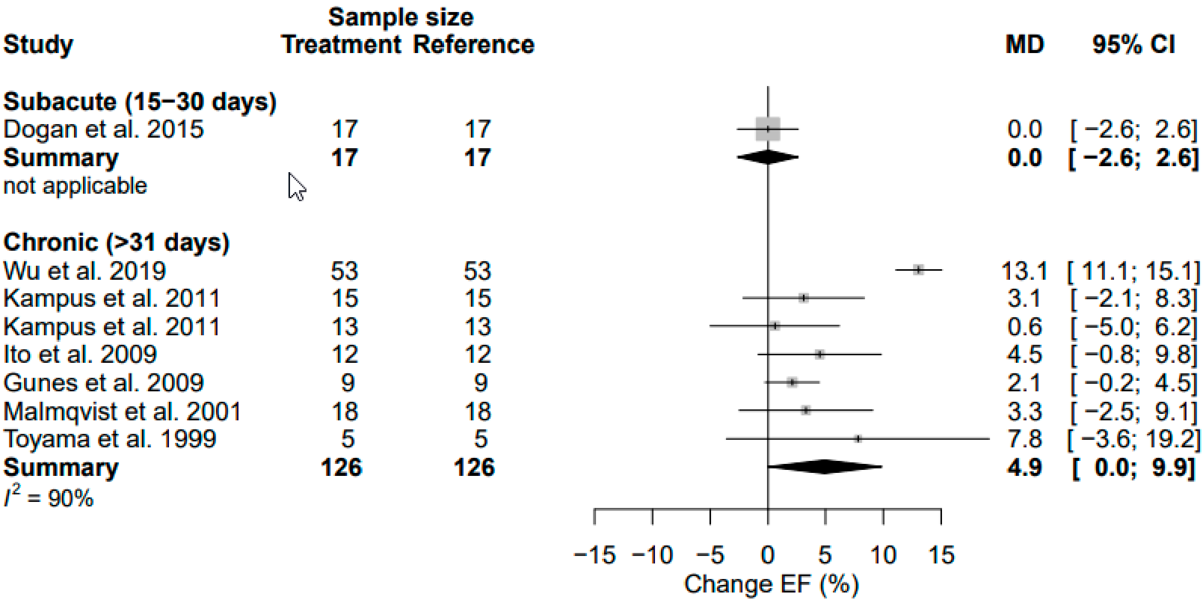

Figure S12 Forest plot of left ventricular ejection fraction (LVEF) change in % after sub-acute and chronic be-ta-blocker use compared to baseline for females. MD = mean difference.

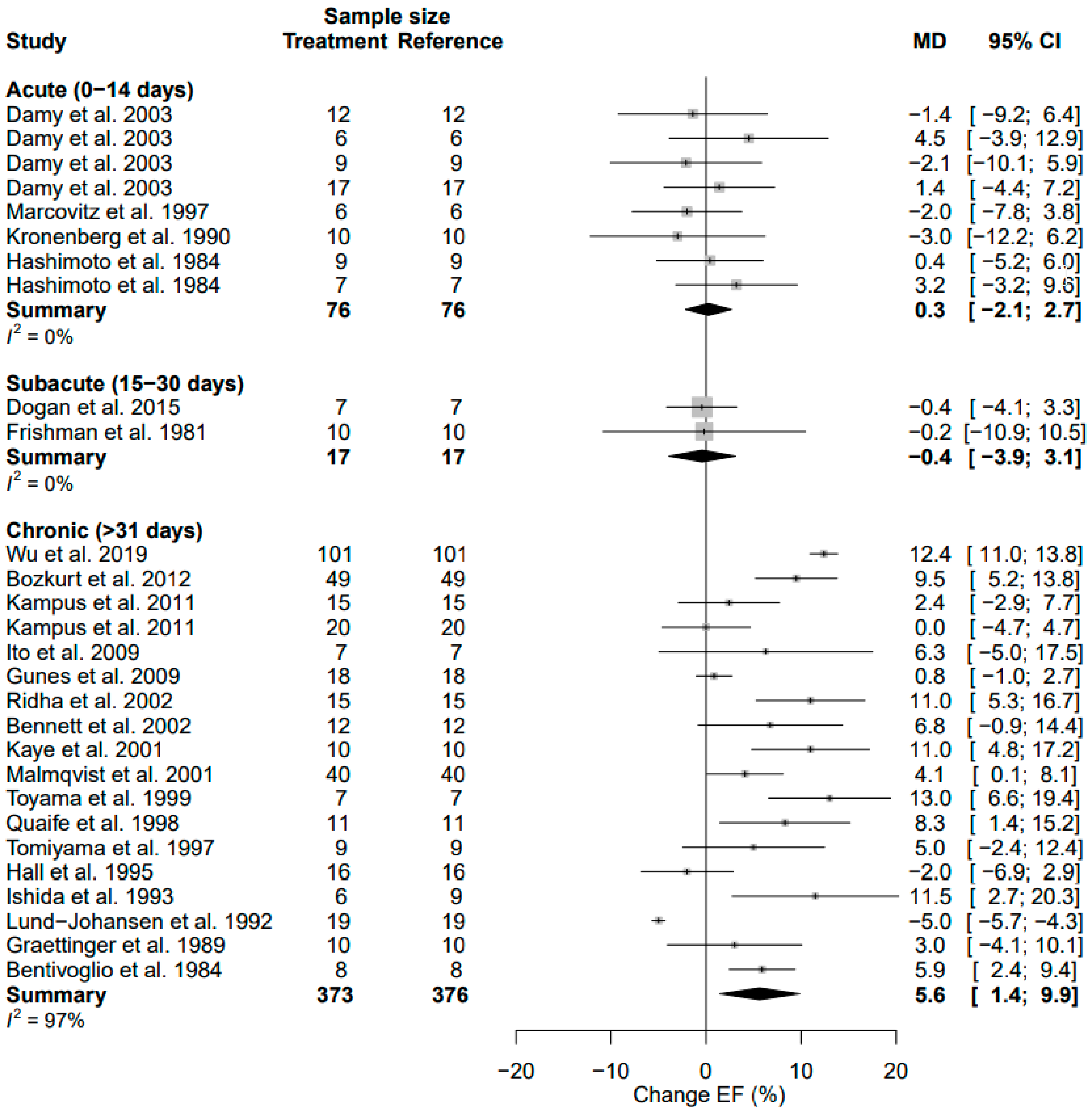

Figure S13 Forest plot of left ventricular ejection fraction (LVEF) change in % after acute, sub-acute and chronic beta-blocker use compared to baseline for males. MD = mean difference.

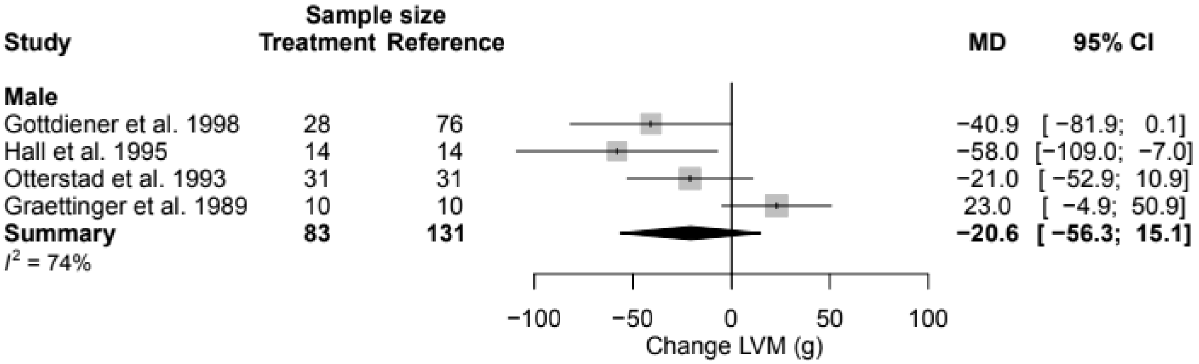

Figure S14 Forest plot of left ventricular mass (LVM) change in grams after beta-blocker use compared to baseline for females and males. MD = mean difference.
